# Frequent Outpatient Visits Prevent Exacerbation of Chronic Obstructive Pulmonary Disease

**DOI:** 10.1038/s41598-020-63064-x

**Published:** 2020-04-08

**Authors:** Hye Jung Park, Min Kwang Byun, Taehee Kim, Chin Kook Rhee, Kyungjoo Kim, Bo Yeon Kim, Sang In Ahn, Yon U Jo, Kwang-Ha Yoo

**Affiliations:** 1 Department of Internal Medicine, Gangnam Severance Hospital, Yonsei University College of Medicine, Seoul, Korea; 2 Division of Pulmonary, Allergy and Critical Care Medicine, Department of Internal Medicine, Seoul St Mary’s Hospital, College of Medicine, The Catholic University of Korea, Seocho, South Korea; 3 Healthcare Review and Assessment Committee, Health Insurance Review & Assessment Service, Seoul, Korea; 4 Division of Chronic Disease Assessment, Health Insurance Review & Assessment Service, Seoul, Korea; 5 Division of Quality Assessment Management, Health Insurance Review & Assessment Service, Seoul, Korea; 6 Division of Pulmonary, Allergy and Critical Care Medicine, Department of Internal Medicine, Konkuk University School of Medicine, Seoul, South Korea

**Keywords:** Risk factors, Chronic obstructive pulmonary disease

## Abstract

Chronic obstructive pulmonary disease (COPD) is a chronic inflammatory airway disease requiring frequent outpatient visits and lifelong management. We aimed to evaluate the roles of frequent outpatient visits in prognosis of COPD. We used claims data in the national medical insurance review system provided by the Health Insurance Review and Assessment Service of Korea from May 1, 2014 to April 30, 2015. A definition of COPD was used based on the diagnosis code and medication. Frequent visitors were defined as subjects who visited the outpatient clinic for COPD three or more times per year. Among 159,025 subjects, 117,483 (73.9%) were classified as frequent visitors. Frequent visitors underwent pulmonary function tests and used various inhalers more often than did infrequent visitors. The rates of COPD exacerbation requiring admission to a general ward, emergency room, or intensive care unit were significantly lower in frequent visitors than in infrequent visitors. In multivariable analysis, frequent visits were identified as an independent factor preventing COPD exacerbation that required admission to a ward (odds ratio [OR], 0.387), emergency room, (OR, 0.558), or intensive care unit (OR, 0.39) (all *P* < 0.001). In conclusion, we showed frequent outpatient visits reduce the risk of COPD exacerbation by 45–60%.

## Introduction

Chronic obstructive pulmonary disease (COPD) is a chronic inflammatory disease of the airways that requires lifelong management^1^. A report from the Global Initiative for Chronic Obstructive Lung Disease have described routine patient follow-up as essential^2^. Lung function may worsen over time because of the natural history of COPD, so regular visits are needed to ensure frequent pulmonary function tests^3^. Symptoms and history of exacerbations should be monitored at these visits to adjust treatment, identify complications or comorbidities, and prevent acute exacerbations^4,5^. Frequent outpatient visits also offer patients an opportunity to improve their skills using inhalers, increase their knowledge of rehabilitation and nutritional support, and receive vaccination, all of which improve the clinical outcomes of COPD^6–9^. However, although there is good reason to believe that frequent outpatient visits have a prognostic benefit in COPD, there is insufficient scientific evidence to support this assumption.

Until now, whether or not frequent outpatient visits have a prognostic benefit in COPD and how often patients should visit a hospital outpatient clinic to prevent exacerbations has never been investigated. The aim of this study was to determine whether or not frequent outpatient visits reduce the risk of exacerbation of COPD using data from a Korean national cohort study.

## Methods

### Ethics statement

The study was approved by the National Evidence-Based Healthcare Collaborating Agency Ethics Committee. The need for informed consent was waived by the institutional review board of Gangnam Severance Hospital, Yonsei University Health System (approval number 3-2018-0337). All aspects of the study were performed in accordance with relevant guidelines and regulations.

### Data sources

South Korea has adopted a single mandatory government-established health care insurance system, and the Health Insurance Review and Assessment Service (HIRA) is the agency that evaluates all medical claims data in South Korea. The HIRA has now accumulated all medical records in South Korea and covers the country’s entire population (>50 million)^10^. The HIRA data have been extensively described in previous studies^11,12^. In this study, we retrospectively analysed the data registered in the HIRA database between May 1, 2014 and April 30, 2015.

### Study population

Patients with COPD were defined as those who met the criteria used in a previous report that included HIRA data^13^. This definition is concordant with that used by the HIRA in its COPD quality evaluation program. The criteria used were as follows: age ≥40 years; ICD-10 codes for COPD or emphysema (J43.0×-J44.x, except for J43.0 as primary or secondary [within fourth position’ diagnosis]); and use of more than one COPD medication at least twice a year.

### Definition of terms

Frequent visitors were defined as those who visited a hospital outpatient clinic for follow-up of COPD three or more times per year regardless of the interval between visits and the site, and others were classified as infrequent visitors. The Charlson Comorbidity Index (CCI), which facilitates prediction of the prognosis and mortality, was calculated as previously described^14,15^. Exacerbation of COPD requiring hospital admission was defined as an admission to a general ward, emergency room (ER), or intensive care unit (ICU) with a diagnosis of COPD as the principal or first additional diagnosis.

### Statistical analysis

The *t-*test and chi-square test were used to identify differences in continuous and categorical variables, respectively, between frequent and infrequent visitors. Univariable and multivariable logistic regression analyses were used to identify significant risk factors for exacerbation of COPD. A cumulative risk ratio graph was drawn using the Kaplan-Meier method. A Cox regression model was used to identify significant risk factors for exacerbation of COPD over time with adjustment for various factors. All statistical analyses were performed using SPSS for Windows (version 18.0; IBM Corp., Armonk, NY). A *P*-value <0.05 was considered statistically significant.

## Results

### Subject demographics

We classified the 159,025 patients with COPD registered in the HIRA as infrequent visitors (n = 41,542) or frequent visitors (n = 117,483). The frequent visitor group was significantly older and included more male patients. Frequent visitors were more likely than infrequent visitors to visit a primary care facility for follow-up. Frequent visitors had more comorbidity in the form of osteoporosis, depressive disorder, arthritis, diabetes mellitus, hypertension, and allergic rhinitis, whereas pneumothorax and congestive heart failure was more common in infrequent visitors. However, there was no significant between-group difference in the CCI value. The proportion of patients admitted with an exacerbation of COPD in the previous year was significantly higher in the frequent visitor group than in the infrequent visitor group (9.2% vs 7.2%, *P* < 0.001; Table 1).

**Table 1:** Demographics of patients attending frequently and infrequently for management of chronic obstructive pulmonary disease.

Parameter	Infrequent visitor group	Frequent visitor group	*P*-value
Age, years (mean and SD)	70.8 ± 10.9	71.1 ± 9.9	<0.001
Male sex, %	64.6	74.8	<0.001
With medical aid insurance, %	12.7	16.0	<0.001
Type of hospital, %			
Primary care	60.0	70.0	<0.001
Secondary	15.4	11.9	<0.001
Tertiary	31.9	29.6	<0.001
Comorbidity, %			
**Ischemic heart disease**	**1.5**	**1.6**	**0.449**
Osteoporosis	2.3	2.7	<0.001
Depressive disorder	1.0	1.6	<0.001
Arthritis	0.5	0.9	<0.001
Diabetes mellitus	8.5	9.2	<0.001
Pneumothorax	0.9	0.8	<0.001
Congestive heart failure	5.6	4.8	<0.001
Hypertension	15.8	22.1	<0.001
**Anaemia**	**1.8**	**1.7**	**0.431**
Allergic rhinitis	27.6	38.4	<0.001
**CCI (mean and SD)**	**1.4** ± **0.8**	**1.2** ± **0.8**	**0.846**
Admission with exacerbated COPD in previous year, %	7.2	9.2	<0.001
Total number of subjects	41,542	117,483	

CCI, Charlson Comorbidity Index; COPD, chronic obstructive pulmonary disease; CT, computed tomography; SD, standard deviation

Presented as bold in statistically insignificant value.

### Test performed, medications prescribed, and health care utilization

Radiographic and computed tomography examinations were performed less often in frequent visitors than in infrequent visitors. However, frequent visitors underwent pulmonary function tests more often than did infrequent visitors. All medications were prescribed more often for frequent visitors than for infrequent visitors The mean number of outpatient visits was significantly higher in the frequent visitor group than in the infrequent visitor group (6.81 ± 6.21 vs 1.74 ± 0.59, *P* < 0.001). The mean number of inpatient visits was significantly lower in the frequent visitor group than in the infrequent visitor group (0.20 ± 0.73 vs 0.43 ± 1.01, *P* < 0.001), as was the proportion of subjects ever admitted to a general ward (11.8% vs 23.8%, *P* < 0.001). The mean numbers of ER and ICU admissions were also significantly lower in the frequent visitor group than in the infrequent visitor group. Furthermore, the percentages of subjects ever admitted to an ER or ICU were significantly lower in the frequent visitor group than in the infrequent visitor group. (Table 2).

**Table 2:** Health care utilization and medications prescribed according to study group.

Parameter	Infrequent visitor group	Frequent visitor group	*P*-value
***Tests performed, %***			
Chest radiography	14.0	13.1	<0.001
Chest CT scan	1.2	0.6	<0.001
Pulmonary function tests	33.2	45.7	<0.001
***Prescribed medication, %***			
Systemic beta-agonist	26.0	29.9	<0.001
Theophylline	56.9	66.0	<0.001
LABA	6.3	10.5	<0.001
LAMA	30.6	50.1	<0.001
LABA/LAMA	0.0	0.1	0.008
SABA	29.2	31.1	<0.001
SAMA	13.5	10.6	<0.001
ICS	12.6	15.0	<0.001
ICS/LABA	29.1	43.9	<0.001
PDE-4 inhibitors	1.0	3.3	<0.001
***Medical service utilization***			
[Outpatient visit]			
Mean number of outpatient visits	1.74 ± 0.59	6.81 ± 6.21	<0.001
Outpatient visit, %	92.1	100.0	<0.001
[Inpatient admission]			
Mean number of admissions	0.43 ± 1.02	0.20 ± 0.73	<0.001
Admission to a general ward, %	23.8	11.8	<0.001
[ER admission]			
Mean number of admissions	0.13 ± 0.44	0.10 ± 0.59	<0.001
Admission to ER, %	10.0	6.9	0.001
[ICU admission]			
Mean number of admissions	0.05 ± 0.29	0.02 ± 0.17	<0.001
Admission to ICU, %	3.5	1.6	<0.001

ER, emergency room; ICS, inhaled corticosteroid; ICU, intensive care unit; LABA, long-acting beta-2 agonist; LAMA, long-acting muscarinic antagonist; PDE-4, phosphodiesterase-4; SABA, short-acting beta-2 agonist; SAMA, short-acting muscarinic antagonist; SD, standard deviation.

Presented as bold in statistically insignificant value.

### Significant risk factors for exacerbation of COPD requiring admission to a general ward

Older age, female sex, having medical aid insurance, attending a low-grade care facility, a high CCI, and admission with an exacerbation of COPD in the previous year were significant risk factors for an inpatient admission in the univariable and multivariable analyses. Frequent visiting was a protective factor in univariable analysis (odds ratio [OR] 0.428; 95% confidence interval [CI] 0.416–0.441; *P* < 0.001). After adjustment for the above-mentioned confounding factors, frequent visiting significantly reduced the likelihood of a COPD exacerbation requiring admission to a general ward (OR 0.294; 95% CI 0.284–0.304; *P* < 0.001, Table 3).

**Table 3:** Significant risk factors for exacerbation of chronic obstructive pulmonary disease in a logistic regression model.

Parameter	Univariable analysis		Multivariable analysis	
	OR (95% CI)	*P*-value	OR (95% CI)	*P*-value
***COPD exacerbation requiring general ward admission***				
Age	1.027 (1.026–1.029)	<0.001	1.019 (1.017–1.020)	<0.001
Male sex	0.896 (0.869–0.923)	<0.001	0.760 (0.733–0.788)	<0.001
Medical aid insurance	1.438 (1.388–1.490)	<0.001	1.159 (1.111–1.209)	<0.001
Type of hospital				
Primary care	2.592 (2.502–2.685)	<0.001	11.992 (11.306–12.720)	<0.001
Secondary	3.870 (3.744–4.000)	<0.001	13.182 (12.479–13.926)	<0.001
Tertiary	0.406 (0.391–0.421)	<0.001	1.73 (1.649–1.814)	<0.001
CCI	2.095 (2.064–2.126)	<0.001	1.931 (1.900–1.963)	<0.001
Admission with exacerbated COPD in previous year	5.819 (5.607–6.039)	<0.001	4.659(4.463–4.864)	<0.001
Frequent visitor	0.428 (0.416–0.441)	<0.001	0.294(0.284–0.304)	<0.001
***COPD exacerbation requiring ER admission***				
Age	1.030 (1.028–1.032)	<0.001	1.023 (1.021–1.025)	<0.001
**Male sex**	**1.206 (1.155–1.258)**	**<0.001**	**0.97 (0.928–1.020)**	**0.250**
Medical aid insurance	1.403 (1.339–1.471)	<0.001	1.175 (1.116–1.237)	<0.001
Type of hospital				
Primary care	11.824 (10.833–12.906)	<0.001	24.448 (22.179–26.948)	<0.001
Secondary	1.471 (1.400–1.545)	<0.001	3.400 (3.194–3.612)	<0.001
Tertiary	0.411 (0.391–0.432)	<0.001	1.713 (1.617–1.816)	<0.001
CCI	1.795 (1.766–1.825)	<0.001	1.592 (1.563–1.621)	<0.001
Admission with exacerbated COPD in previous year	4.730 (4.523–4.946)	<0.001	3.313 (3.15503.479)	<0.001
Frequent visitor	0.663 (0.638–0.690)	<0.001	0.515 (0.493–0.538)	<0.001
***COPD exacerbation requiring ICU admission***				
Age	1.041 (1.037–1.045)	<0.001	1.033 (1.029–1.037)	<0.001
Male sex	1.045 (0.967–1.129)	0.268	0.872 (0.804–0.947)	0.001
**Medical aid insurance**	**1.274 (1.165–1.392)**	**<0.001**	**1.028 (0.936–1.130)**	**0.558**
Type of hospital				
Primary care	35.315 (26.394–47.253)	<0.001	54.603 (40.478–73.656)	<0.001
Secondary	1.201 (1.090–1.323)	<0.001	2.093 (1.880–2.329)	<0.001
Tertiary	0.363 (0.329–0.401)	<0.001	1.464 (1.315–1.629)	<0.001
CCI	1.916 (1.868–1.964)	<0.001	1.687 (1.642–1.734)	<0.001
Admission with exacerbated COPD in previous year	4.383 (4.054–4.737)	<0.001	2.840 (2.614–3.08)	<0.001
Frequent visitor	0.449 (0.419–0.481)	<0.001	0.370 (0.343–0.398)	<0.001

CCI, Charlson Comorbidity Index; CI, confidence interval; COPD, chronic obstructive pulmonary disease; ER, emergency room; ICU, intensive care unit; OR, odds ratio.

Presented as bold in statistically insignificant value.

**Figure 1:**
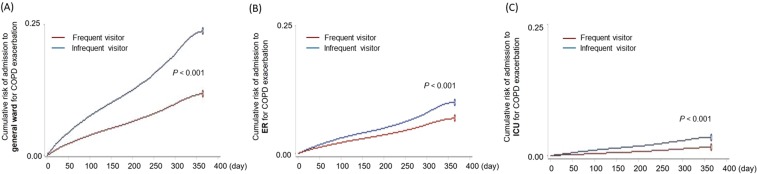
Kaplan-Meier graph showing the cumulative risk of admission to a general ward (a), ER (b), or ICU (C) over time. ER, emergency room; ICU, intensive care unit.

The Kaplan-Meier graph showed a significant difference in the risk of admission over time between the frequent visitor group and the infrequent visitor group (OR 0.464; 95% CI 0.453–0.477; *P* < 0.001; Fig. 1A). Cox regression analysis showed a significant reduction in the risk of COPD exacerbation requiring admission to a general ward in the frequent visitor group after adjustment for the confounding factors (OR 0.387; 95% CI 0.377–0.397; *P* < 0.001, Table 4).

**Table 4:** Significant risk factors for exacerbation of chronic obstructive pulmonary disease over time in a Cox regression model.

Parameter	Univariable analysis		Multivariable analysis	
	OR (95% CI)	*P*-value	OR (95% CI)	*P*-value
***COPD exacerbation requiring general ward admission***				
Age	1.025 (1.024–1.026)	<0.001	1.014 (1.013–1.015)	<0.001
Male sex	0.906 (0.881–0.932)	<0.001	0.834 (0.810–0.858)	<0.001
Medical aid insurance	1.407 (1.362–1.453)	<0.001	1.147 (1.110–1.185)	<0.001
Type of hospital				
Primary care	2.434 (2.354–2.517)	<0.001	4.84 (4.650–5.038)	<0.001
Secondary	3.303 (3.212–3.396)	<0.001	4.921 (4.767–5.081)	<0.001
Tertiary	0.428 (0.414–0.443)	<0.001	1.252 (1.206–1.300)	<0.001
CCI	1.756 (1.739–1.772)	<0.001	1.518 (1.503–1.533)	<0.001
Admission with exacerbated COPD in previous year	4.764 (4.627–4.905)	<0.001	3.193 (3.098–3.291)	<0.001
Frequent visitor	0.464 (0.453–0.477)	<0.001	0.387 (0.377–0.397)	<0.001
***COPD exacerbation requiring ER admission***				
Age	1.028 (1.026–1.030)	<0.001	1.021 (1.019–1.023)	<0.001
**Male sex**	**1.198 (1.150–1.249)**	**<0.001**	**0.982 (0.942–1.024)**	**0.398**
Medical aid insurance	1.387 (1.326–1.450)	<0.001	1.153 (1.102–1.207)	<0.001
Type of hospital				
Primary care	11.189 (10.259–12.204)	<0.001	18.308 (16.694–20.079)	<0.001
Secondary	1.448 (1.382–1.518)	<0.001	2.577 (2.453–2.708)	<0.001
Tertiary	0.422 (0.402–0.443)	<0.001	1.522 (1.475–1.632)	<0.001
CCI	1.664 (1.642–1.685)	<0.001	1.442 (1.422–1.462)	<0.001
Admission with exacerbated COPD in previous year	4.325 (4.155–4.502)	<0.001	2.831 (2.717–2.951)	<0.001
Frequent visitor	0.676 (0.651–0.702)	<0.001	0.558 (0.537–0.579)	<0.001
***COPD exacerbation requiring ICU admission***				
Age	1.041 (1.037–1.044)	<0.001	1.031 (1.027–1.035)	<0.001
Male sex	1.044 (0.967–1.127)	0.276	0.871 (0.806–0.942)	0.001
**Medical aid insurance**	**1.270 (1.163–1.387)**	**<0.001**	**1.039 (0.951–1.136)**	**0.399**
Type of hospital				
Primary care	34.789 (26.005–46.540)	<0.001	49.947 (37.081–67.278)	<0.001
Secondary	1.198 (1.089–1.319)	<0.001	1.916 (1.735–2.117)	<0.001
Tertiary	0.366 (0.332–0.404)	<0.001	1.432 (1.294–1.584)	<0.001
CCI	1.831 (1.791–1.871)	<0.001	1.568 (1.533–1.603)	<0.001
Admission with exacerbated COPD in previous year	4.284 (3.971–4.621)	<0.001	2.658 (2.457–2.876)	<0.001
Frequent visitor	0.453 (0.423–0.485)	<0.001	0.391 (0.365–0.420)	<0.001

CCI, Charlson Comorbidity Index; CI, confidence interval; COPD, chronic obstructive pulmonary disease; ER, emergency room; ICU, intensive care unit; OR, odds ratio.

Presented as bold in statistically insignificant value.

### Significant risk factors for exacerbation of COPD requiring admission to an emergency room

The risk factors for exacerbation of COPD requiring admission to an ER were similar to those for inpatient admission. Frequent visitors had a significantly lower ER admission rate in univariable analysis (OR 0.663; 95% CI 0.638–0–690; *P* < 0.001) and multivariable analysis (OR 0.515; 95% CI 0.493–0.538; *P* < 0.001; Table 3).

The Kaplan-Meier graph and Cox regression analysis identified frequent visiting as significantly protective against exacerbation of COPD requiring ER admission after adjustment for the above factors (OR 0.676; 95% CI 0.651–0.702; *P* < 0.001, Fig. 1B) (OR 0.558; 95% CI 0.537–0.579; *P* < 0.001, Table 4).

### Significant risk factors for exacerbation of COPD requiring admission to an ICU

The risk factors for exacerbation of COPD requiring admission to an ICU were also similar to those for inpatient admission The ICU admission rate was significantly lower in the frequent visitor group in univariable analysis (OR 0.449; 95% CI 0.419–0.481; *P* < 0.001) and multivariable analysis (OR 0.370; 95% CI 0.343–0.398; *P* < 0.001, Table 3).

The Kaplan-Meier graph and Cox regression analysis showed that the risk of an ICU admission over time was significantly lower in the frequent visitor group than in the infrequent visitor group (OR 0.453; 95% CI 0.423–0.485; *P* < 0.001, Fig. 1C) (OR 0.391; 95% CI 0.365–0.420; *P* < 0.001, Table 4).

## Discussion

The results of this study show that frequent outpatient visits (three or more times per year) for management of COPD can reduce the risk of an exacerbation requiring admission by 45–60%. The rate of reduction in exacerbation of COPD achieved in the present study is much higher than that reportedly achieved by use of inhalers which is well-known important treatment modality in COPD (15–30%)^16,17^. COPD is a chronic disease that requires patients to understand the nature of their condition, to cooperate with their doctors, and to manage their disease lifelong. Frequent outpatient visits offer the opportunity for patients to have their lung function and symptom control checked, to improve their skills in using inhalers, and to have their treatment adjusted to prevent acute exacerbation. This study confirmed that frequent visitors had more frequent pulmonary function tests and were prescribed COPD medications, mainly inhalers, more often compared with infrequent visitors. Frequent checks of disease status and early upstaging of COPD medication might have influenced the prognosis of the patients in our study. Frequent visits can also reflect good adherence to treatment. With these various effects, simply making frequent outpatient visits can greatly improve the prognosis of COPD.

However, frequent outpatient visits could mean not only “good adherence” but also “severe COPD”. The severity of COPD is strongly associated with exacerbation during the previous year, symptom score, and airflow obstruction as confirmed by PFT^18,19^. Due to the inevitable limitation of national big data, symptom score and the results of PFT could not be obtained. In this study, “admission with exacerbated COPD in the previous year” was an important factor for the prognosis of COPD. Therefore, we adjusted this parameter to reflect the severity of COPD. Although this substitution and adjustment is not perfect, the effectiveness of “frequent visit” can be approximated to the effectiveness of “good adherence” in multivariable analysis.

The type of hospital was one of the important factors in the exacerbation of COPD in this study. The proportion of general healthcare providers, specialists of internal medicine, and sub-specialists in pulmonology might vary according to the type of hospital. Therefore, the quality of care can vary according to the type of hospital. A recent study showed that the prescription rate of inhaled bronchodilator, which is a fundamental drug of choice for COPD, was 92.6% and 40.5% in tertiary hospitals and private clinics, respectively^20^. Therefore, we also adjusted this factor, ‘type of hospital’, to reduce the confounding factor in Tables 3, 4.

Until now, there have been no studies of the benefits of frequent outpatient visits in terms of the prognosis and how often patients should visit an outpatient clinic. The findings of a study by Bischoff *et al*. on routine monitoring of COPD in general practice were negative^21^. However, in that study, the frequency of monitoring was determined by general practitioners, and 20% of patients attended for routine monitoring only once a year^21^. Therefore, it is probable that this relatively low frequency of outpatient visits accounts for the negative results. Einarsdottir *et al*. showed that regular primary care visits can prevent hospitalization and mortality in patients with COPD^22^. However, they did not concentrate on the ‘frequency’ of clinical visits; instead, they focused on the effects of ‘regularity’. Moreover, only older individuals (aged ≥ 65 years), who had a variety of chronic respiratory diseases including asthma, were enrolled in that study. In addition, we have previously reported on the benefits of frequent outpatient visits by patients with ‘asthma’ but not COPD^12^. Indeed, the significant contribution of frequent hospital visits to the prognosis of COPD has been demonstrated for the first time in the present study.

Uncontrolled symptoms and a history of exacerbation in the previous year can prompt patients to visit an outpatient clinic more frequently. In this study, most of the medications were more predominantly prescribed to the frequent visitors compared to infrequent visitors. It can mean that frequent visitors have severe symptoms or ‘good adherence’. In addition, exacerbation of COPD in the previous year was also more prevalent in frequent visitors than in infrequent visitors (9.2% vs. 7.2%). We can assume that the frequent visitors represent a more severe status of COPD compared to infrequent visitors. Although frequent visitors have severe COPD, the exacerbation rate in frequent visitors was similar in the following year (11.8%) to that in the previous year (9.2%); however, infrequent visitors showed a marked increase in exacerbation rate in the following year (to 23.8% from 7.2%). Therefore, we can infer that infrequent visitors are at risk of exacerbation of COPD even if they have rarely experienced an exacerbation in the previous year.

Multiple comorbidities can also encourage patients to visit an outpatient clinic frequently. However, in this study, we found that the type of comorbidity tended to differ between the two study groups. Most comorbidities were more prevalent in frequent visitors than in infrequent visitors. However, congestive heart failure and pneumothorax was more prevalent in infrequent visitors than in frequent visitors. The CCI was slightly higher in infrequent visitors than in frequent visitors, but the difference was not statistically significant. Further studies concerning the relationship between comorbidity and hospital visits are needed.

We chose three visits as the cut-off number of outpatient clinic visits per year to classify subjects as frequent or infrequent visitors with following reasons. First, we considered that one or two outpatient visits per year would be too few, considering the negative results in a previous study^21^. Second, another study demonstrated that a cut-off value of three was significant in asthma, which is another representative chronic respiratory disease^12^. Last, in Korea, a COPD quality evaluation program was launched in 2014 to improve the quality of the management protocol implemented by all hospitals in Korea; and that includes frequent outpatient visits (three times or more per year) as an important parameter. We wanted to know whether this parameter is really needed.

The effect of frequent visits on the admission rate for exacerbation of COPD depended on the admission route. In frequent visitors, the rate of admission to a general ward or ICU decreased by 61% but by only 44% for admission to an ER. Acute exacerbation of COPD cannot be entirely prevented. Respiratory infection, a change in climate, increased presence of dust particles, and exposure to noxious gases can exacerbate COPD regardless of whether or not treatment and management is adequate^23^. These factors can affect the likelihood of an ER admission because of their unexpected and sudden nature. An ER admission is usually determined by environmental factors rather than the degree of disease control. A previous study also showed that frequent visits reduced the risk of an ER admission to a similar extent^12^. We suggest that the positive effects of frequent visits on unexpected and sudden events are relatively weak and led to frequent ER admissions.

COPD is highly prevalent and a major cause of death worldwide^24^. However, only a small number (2–3%) of patients with COPD are diagnosed to have this condition by doctors in clinics in Korea^25^, as shown in world-wide^26^. COPD needs sustained follow-up, so primary care has a significant role in its management. This study showed that only 60–70% of patients with COPD visit for primary care, in contrast with patients with asthma, 82% of whom were reported to be reliant on primary care^22^. This suggests that patients with COPD in Korea are more likely to visit a hospital when COPD becomes troublesome. This finding further underscores the need to identify and manage patients with COPD more diligently.

In this study, frequent visitors occupied 73.9% of total COPD patients. We think this proportion will be larger than that observed in other countries. In Korea, medical cost is relatively low, then patients can easily visit hospital. Clinicians should carefully apply the results of this study in their countries, considering these unique characteristics of high prevalence of frequent visitors and the low medical cost in Korea.

The main strength of this study is that that we included almost all patients with COPD in Korea. Considering the prevalence rate (10–15%) and diagnosis rate (2–3%) along with the total population in Korea (50 million), the 159,025 subjects enrolled in this study might cover almost all patients with COPD in Korea^25^. However, the study also has several limitations. First, the operational definition of COPD used in this study might be different from the real-world definition of COPD assessed by pulmonary function tests. Second, the study was conducted in Korea, where relatively low hospital utilization costs may lead patients with COPD to visit hospital more readily and could affect the frequency of outpatient visits and admissions. Third, the cost-effectiveness should be studied. Fourth, other variables that could have effects on exacerbation of COPD including current smoking status^27^ were not evaluated because the HIRA data includes only a limited number of variables. Finally, the management protocol including inhaler training, smoking cessation, and adherence varied according to the institutes.

## Conclusions

Frequent outpatient visits provide an opportunity for patients with COPD to undergo pulmonary function tests more often and to have their inhaler medication adjusted earlier than would be the case if they made infrequent outpatient visits. Frequent outpatient visits can reduce the risk of exacerbations requiring admission by 45–60%. We recommend that patients with COPD should visit at least three times per year to optimise their prognosis.

## Data Availability

The datasets generated during the current study are available from the corresponding author on reasonable request.

## References

[1] Park YB (2018). Revised (2018) COPD Clinical Practice Guideline of the Korean Academy of Tuberculosis and Respiratory Disease: A Summary. Tuberc Respir Dis (Seoul).

[2] Alvar Agusti, B. R. C. *et al*. GOLD Reports 2019, https://goldcopd.org/gold-reports/ assess available at 20th Jan 2019. (2019).

[3] Vestbo J, Lange P (2016). Natural history of COPD: Focusing on change in FEV1. Respirology.

[4] Viniol, C. and Vogelmeier, C. F. Exacerbations of COPD. *Eur Respir Rev* **27**, 10.1183/16000617.0103-2017 (2018). 10.1183/16000617.0103-2017 PMC9488662 29540496

[5] Gardener AC, Ewing G, Kuhn I, Farquhar M (2018). Support needs of patients with COPD: a systematic literature search and narrative review. Int J Chron Obstruct Pulmon Dis.

[6] Melani AS (2011). Inhaler mishandling remains common in real life and is associated with reduced disease control. Respir Med.

[7] Spruit MA (2013). An official American Thoracic Society/European Respiratory Society statement: key concepts and advances in pulmonary rehabilitation. Am J Respir Crit Care Med.

[8] Ferreira IM, Brooks D, White J, Goldstein R (2012). Nutritional supplementation for stable chronic obstructive pulmonary disease. Cochrane Database Syst Rev.

[9] Bekkat-Berkani R (2017). Seasonal influenza vaccination in patients with COPD: a systematic literature review. BMC Pulm Med.

[10] Yang MS (2016). Incidence of Stevens-Johnson Syndrome and Toxic Epidermal Necrolysis: A Nationwide Population-Based Study Using National Health Insurance Database in Korea. PLoS One.

[11] Kim JA, Yoon S, Kim LY, Kim DS (2017). Towards Actualizing the Value Potential of Korea Health Insurance Review and Assessment (HIRA) Data as a Resource for Health Research: Strengths, Limitations, Applications, and Strategies for Optimal Use of HIRA Data. J Korean Med Sci.

[12] Park HJ (2018). Regular follow-up visits reduce the risk for asthma exacerbation requiring admission in Korean adults with asthma. Allergy Asthma Clin Immunol.

[13] Lee J, Lee JH, Kim JA, Rhee CK (2017). Trend of cost and utilization of COPD medication in Korea. Int J Chron Obstruct Pulmon Dis.

[14] Charlson ME, Pompei P, Ales KL, MacKenzie CR (1987). A new method of classifying prognostic comorbidity in longitudinal studies: development and validation. J Chronic Dis.

[15] Song SE (2016). The Prognostic Value of the Charlson’s Comorbidity Index in Patients with Prolonged Acute Mechanical Ventilation: A Single Center Experience. Tuberc Respir Dis (Seoul).

[16] Halpin DM (2016). Effect of tiotropium on COPD exacerbations: A systematic review. Respir Med.

[17] Karbasi-Afshar R, Aslani J, Ghanei M (2014). Efficacy and safety of inhaler steroids in COPD patients: Systematic review and meta-analysis of randomized placebo-controlled trials. Caspian J Intern Med.

[18] Celli BR (2004). The body-mass index, airflow obstruction, dyspnea, and exercise capacity index in chronic obstructive pulmonary disease. N Engl J Med.

[19] Puhan MA (2009). Expansion of the prognostic assessment of patients with chronic obstructive pulmonary disease: the updated BODE index and the ADO index. Lancet.

[20] Chung SM, Lee SY (2017). Evaluation of Appropriate Management of Chronic Obstructive Pulmonary Disease in Korea: Based on Health Insurance Review and Assessment Service (HIRA) Claims. Tuberc Respir Dis (Seoul).

[21] Bischoff EW (2012). Comprehensive self management and routine monitoring in chronic obstructive pulmonary disease patients in general practice: randomised controlled trial. BMJ.

[22] Einarsdottir K, Preen DB, Emery JD, Kelman C, Holman CD (2010). Regular primary care lowers hospitalisation risk and mortality in seniors with chronic respiratory diseases. J Gen Intern Med.

[23] Miravitlles M, D’Urzo A, Singh D, Koblizek V (2016). Pharmacological strategies to reduce exacerbation risk in COPD: a narrative review. Respir Res.

[24] Brandsma, C. A. *et al*. Lung ageing and COPD: is there a role for ageing in abnormal tissue repair? *Eur Respir Rev* **26**, 10.1183/16000617.0073-2017 (2017). 10.1183/16000617.0073-2017 PMC9488745 29212834

[25] Hwang YI, Park YB, Yoo KH (2017). Recent Trends in the Prevalence of Chronic Obstructive Pulmonary Disease in Korea. Tuberc Respir Dis (Seoul).

[26] Park HJ (2018). Significant predictors of medically diagnosed chronic obstructive pulmonary disease in patients with preserved ratio impaired spirometry: a 3-year cohort study. Respir Res.

[27] Mantero M (2017). Acute exacerbations of COPD: risk factors for failure and relapse. Int J Chron Obstruct Pulmon Dis.

